# Cu-based metal–organic framework HKUST-1 as effective catalyst for highly sensitive determination of ascorbic acid

**DOI:** 10.1039/d0ra01260b

**Published:** 2020-06-16

**Authors:** Tianyang Shen, Tianchen Liu, Hanqi Mo, Zichen Yuan, Feng Cui, Yixiang Jin, Xiaojun Chen

**Affiliations:** College of Chemistry and Molecular Engineering, Nanjing Tech University Nanjing 211800 People's Republic of China chenxj@njtech.edu.cn; College of Overseas Education, Nanjing Tech University Nanjing 211800 People's Republic of China; Nanjing Foreign Language School Nanjing 210018 People's Republic of China; Nanjing No. 1 Middle School Nanjing 210001 People's Republic of China

## Abstract

In this work, a Cu-based nanosheet metal–organic framework (MOF), HKUST-1, was synthesised using a solvent method at room temperature. Its morphology, structure and composition were characterised by scanning electron microscopy (SEM), transmission electron microscopy (TEM), atomic force microscopy (AFM), powder X-ray diffraction (XRD), Fourier transform infrared (FTIR), Raman spectroscopy, nitrogen adsorption and desorption isotherms, energy dispersive X-ray spectroscopy (EDS) and elemental analysis (EA). This material was then loaded onto the surface of an indium tin oxide (ITO) electrode to catalyse the electrochemical oxidation of ascorbic acid (AA). An equal-electron-equal-proton reaction was deduced from the pH investigation, and a diffusion-controlled process was reinforced by the dynamics study. Under optimal conditions, the oxidation peak current at +0.02 V displayed a linear relationship with the concentration of AA within the ranges of 0.01–25 and 25–265 mM, respectively. The limit of detection (LOD) was 3 μM at S/N of 3. The superb response could be ascribed to the porous nanosheet structure of HKUST-1, which enhanced both the effective surface area and the electron transfer ability significantly. Moreover, the novel AA sensor demonstrated good reproducibility, favourable stability and high sensitivity towards glucose, uric acid (UA), dopamine (DA) and several amino acids. It was also successfully applied to the real sample testing of various AA-containing tablets.

## Abstract 2

In this work, a Cu-based nanosheet metal–organic framework (MOF), HKUST-1, was synthesised using a solvent method at room temperature and it demonstrated high capability and sensitivity towards the oxidation of ascorbic acid (AA).

## Graphical abstract

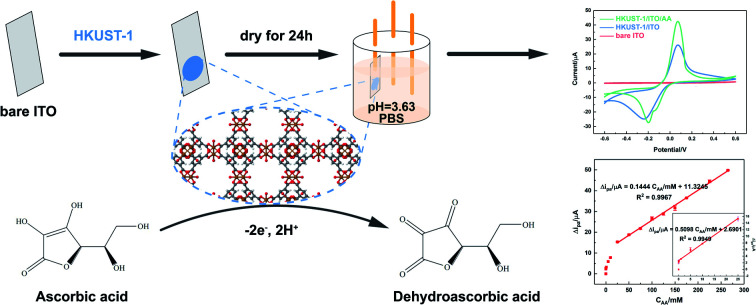

## 1. Introduction

Ascorbic acid (AA), which is also known as vitamin C, is a polyhydroxy compound with a similar structure to glucose.^1^ It is capable of being reduced,^2,3^ and thus is widely used as a natural antioxidant in food,^4^ juice,^5^ medicine^3^ and cosmetics.^6^ It is also an important water-soluble vitamin, which exists extensively as a highly active species participating in the metabolic processes of many creatures,^7^ fresh fruit and vegetables.^5^ Recently, due to the crucial functions in free radical scavenging,^2–4^ cell development and therapeutic areas, such as wound healing, preventing cancer and enhancing immunity,^8^ AA has continuously attracted the public's interest. It was reported that an AA shortage could lead to the symptoms of scurvy,^9^ however, exaggerated amounts could induce stomach convulsions.^10^ Therefore, the determination of AA concentration is of great use, which could be considered as an important physiological indicator for anti-aging.^11–13^ Additionally, because AA is in a millimole or even smaller scale, particularly in human bodies, novel facile and rapid methods contributing to selective and sensitive detection are required. This is exactly significant not only for monitoring human metabolism, but also for the supervision of food, drugs and dietary supplement.^14^

Nowadays, diverse methods have been developed to improve the detection of AA, including ultra- and high-performance liquid chromatography (UPLC or HPLC),^15^ capillary electrophoresis,^16^ fluorescence spectroscopy^17^ and UV-Vis spectroscopy.^18^ Beyond these, electrochemical techniques are often applied, which are easy to implement and not expensive.^19^ With modified electrode surface, the electrochemical response is promoted enormously, and the lower limit of detection (LOD) and wider linear range are accessed.^20^ Nevertheless, the sensitivity and reproducibility usually tend to be the issue because of interference from other biological molecules like dopamine (DA) and uric acid (UA), leading to the challenge of employment in food, drug or real sample analysis.^20^

Recently, many nanomaterials, such as graphene, carbon nanotubes (CNTs)^21^ and nanoparticles (NPs),^22^ have been developed and applied into electrochemical areas. Metal–organic frameworks (MOFs) are novel functional materials composed of repeated spatial or planar patterns of metal ions coordinated to organic ligands through covalent bonds. They have attracted increasing attention owing to the critical role in the field of molecular adsorption,^23^ carbon capture,^24^ compound separation,^25^ supercapacitors,^26^ efficient sensing^27^ and catalysis^28,29^ due to adjustable porous structures and superhigh specific areas. In electrochemical analysis, design and synthesis of different types of MOFs has become an appealing domain to study. It is reported that metal–organic frameworks are well electrochemically active to the oxidation of some small molecules such as glucose,^30^ nitrite,^31^ ethanol,^32^ hydrazine^33^ and dihydroxybenzene isomers,^34^ and the redox process of NADH^35^ and H_2_O_2_.^30,36,37^ However, MOFs are rare as electrochemical sensors compared to others, because organic coordinates are normally bad conductors and only the metal sites are conductive.^37,38^ Hence, it is challenging to prepare highly conductive electrochemical sensors based on MOFs.

To the best of our knowledge, none study has been reported so far on determining the concentration of AA using MOFs-modified electrodes. In this work, the prevalent HKUST-1 framework (also known as MOF-199 or Cu-BTC) was assembled at room temperature and loaded on the indium tin oxide (ITO) electrodes to investigate the capability of electrochemical oxidation of AA. Both the metallic Cu ion centre and the layered structure of HKUST-1 enhanced the electron transfer greatly, improving the catalytic current dramatically. The reaction dynamic was studied to learn the kinetic parameters and the mechanism of the process. The repeatability, stability and anti-interference ability of the proposed AA sensor were also evaluated. Eventually, the application potential of the novel sensor was evidenced by testing AA content in several commercial healthcare tablets as real samples. The current research provides a new perspective for chemical sensor construction based on MOFs platform.

## 2. Experimental

### 2.1. Chemicals

Copper(ii) acetate monohydrate (Cu(CH_3_COO)_2_·H_2_O), 1,3,5-benzenetricarboxylic acid (H_3_BTC), ethanol, acetone, cyclohexane, triethylamine, dimethylformamide (DMF), phosphoric acid (H_3_PO_4_), monosodium phosphate (NaH_2_PO_4_), disodium phosphate (Na_2_HPO_4_), potassium chloride (KCl), magnesium chloride (MgCl_2_), potassium ferrocyanide (K_4_[Fe(CN)_6_]), potassium ferricyanide (K_3_[Fe(CN)_6_]), AA, uric acid (UA), dopamine (DA), glucose, glycine (Gly), l-methionine (l-Met), l-glutamic acid (l-Glu), tryptophan (Trp), l-cysteine (l-Cys), cystine (Cys) and tyrosine (Tyr). Real AA samples, including vitamin C honeysuckle pills, vitamin C buccal tablets for children and multi-vitamin effervescent tablets, were purchased from market. The water used in this work was double distilled water (DDW). The phosphate buffered solution (PBS) was prepared as 0.1 M at pH 6.5 by dissolving 2.1373 g NaH_2_PO_4_ and 2.2560 g Na_2_HPO_4_ in 200 mL water, and was acidified to the desired values by adding 0.2 M of H_3_PO_4_. All chemicals were of analytical grade and used as received.

### 2.2. Apparatus

The morphology of HKUST-1 was studied by scanning electron microscopy (SEM, Hitachi S4800) and transmission electron microscopy (TEM, JEOL JEM-200CX). The thickness was measured by atomic force microscopy (AFM, Bruker, Dimension Icon). The elemental composition analysis was investigated by energy dispersive X-ray spectroscopy (EDS Falcon 60S, EDAX Inc.) and CHNS/O elemental analyzer (Elementar Vario EL Cube). To characterise the crystal structure of HKUST-1, the power X-ray diffraction (XRD) pattern was performed by Philips X'Pert X-ray diffractometer with Cu Kα X-ray source. The bonding and functional groups were recorded using Fourier-transform infrared spectra (Nicolet iS 10 FT-IR spectrophotometer, Thermo Fisher Scientific Inc., USA) and Raman spectra (DXR2 Raman Microscope, Thermo Fisher Scientific Inc., USA). The nitrogen sorption and desorption isotherms were measured with a Micromeritics ASAP 2020 analyser. The specific surface area of HKUST-1 was determined using the standard Brunauer–Emmett–Teller (BET) method, while the pore size distribution was calculated by the Barrett–Joyner–Halenda (BJH) method.

All the cyclic voltammetry (CV) and differential pulse voltammetry (DPV) measurements were implemented on the electrochemical workstation (CHI650E, Shanghai Chenhua Instrument Co., China). A typical three-electrode system was applied in 0.1 M PBS at room temperature, which was composed of a HKUST-1/ITO working electrode, an Ag/AgCl reference electrode and a platinum wire auxiliary electrode.

### 2.3. Preparation of HKUST-1

HKUST-1 was synthesised at room temperature as previous reports.^38,39^ 0.60 g of Cu(CH_3_COO)_2_·H_2_O was dissolved in 50 mL of water and 0.42 g of H_3_BTC was dissolved in 50 mL of ethanol, respectively. After they were completely mixed, another mixture of 10 mL of cyclohexane and 0.85 mL of triethylamine was added dropwise. Then, blue solids formed and precipitated afterwards. The reaction was allowed to stand for 24 h before the solids were centrifuged and washed with DDW and ethanol, sequentially. The obtained product was dried in vacuum for 12 h at 50 °C.

### 2.4. Fabrication of HKUST-1 modified ITO electrode

Typically, 10 mg of HKUST-1 was dispersed into 5 mL of DMF to form a 2.0 mg mL^−1^ stable suspension. All ITO electrodes were washed by acetone followed by ethanol and DDW before use. After drying with nitrogen, 7.0 μL of the suspension was dropped onto ITO surface with the controlled geometric diameter of 1.0 mm and then dried in air for use.^40^

## 3. Results and discussion

### 3.1. Morphology characterisation of HKUST-1

**Fig. 1:**
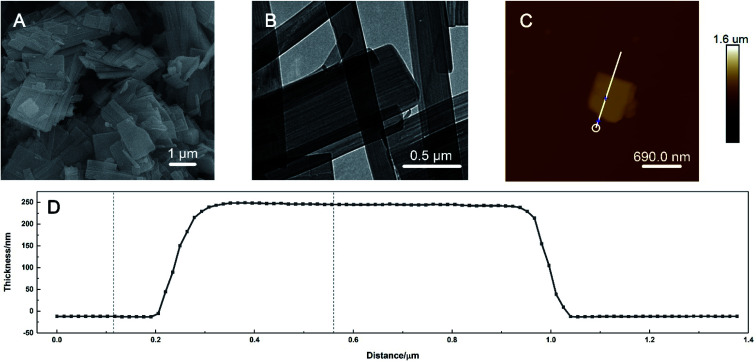
(A) SEM, (B) TEM, (C) AFM images and (D) the corresponding thickness profile of the prepared HKUST-1.

The morphology of the prepared HKUST-1 was characterised by SEM, TEM and AFM. As shown in Fig. 1A, the SEM image exhibited that the shape of the material was irregular-layered sheets with smooth surfaces. The length of the sheets extended to the micrometre scale, as seen in the TEM image in Fig. 1B. The large space between the sheets increased the specific area greatly, which would improve the electron-transfer efficiency when HKUST-1 nanosheets were loaded on the ITO surface. AFM was utilized to measure the average thickness of the sheets. For a specific single layer shown in Fig. 1C, the prepared HKUST-1 sheet has a planar and homogeneous morphology with the thickness of about 254 nm (Fig. 1D).

### 3.2. Structure and composition of HKUST-1

**Fig. 2:**
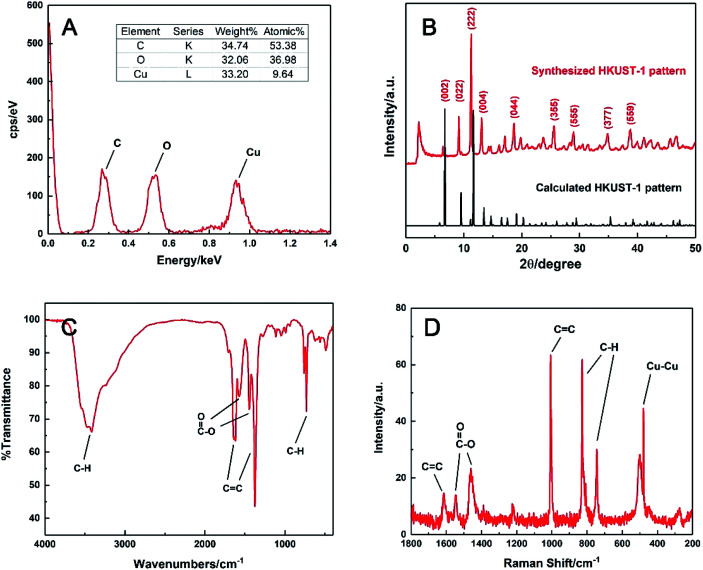
(A) EDS spectrum, (B) experimental XRD pattern (red line) and the simulated curve (black line), (C) FTIR and (D) Raman spectra of the synthesised HKUST-1.

The chemical formula was speculated by EDS analysis in Fig. 2A. It was obvious that the molar ratio of the elements C, O and Cu in the prepared HKUST-1 was 53.38 : 36.98 : 9.64, which were about 6 : 4 : 1. This suggested that this MOF had a formula of Cu_3_(C_9_H_3_O_6_)_2_, meaning that every three Cu(ii) could coordinate with two BTC linkers on average during precipitation. This was supported by elemental analysis as well, and it gave the exact mass percentages of C, H and O in the sample are 32.77%, 1.04% and 32.54%, which is close to the molar ratio of 3 : 1 : 2 and consistent with the formula reported before.^38^

The powder XRD pattern of the synthesised material was compared with the simulated one in Crystal Diffract 6.7 version.^41,42^ As shown in Fig. 2B, the strong peaks at 2*θ* of 6.38, 9.14, 11.26 and 13.08° were identical to the simulated ones, corresponding to the regular (002), (022), (222) and (004) planes of HKUST-1, respectively. Other weak peaks at 2*θ* of 18.66, 25.58, 28.94, 34.78 and 38.78° also matched well with the planes of (044), (355), (555), (377) and (559), respectively. The result confirmed that the prepared HKUST-1 possessed a similar structure to the reported data.^42^

Moreover, the main functional groups of HKUST-1 were characterised *via* FTIR and Raman spectroscopy. In Fig. 2C, the C–H (3400 cm^−1^) and the C=C stretching of benzene ring (1619 and 1375 cm^−1^), the asymmetric and symmetric vibration modes of C=O (1571 and 1446 cm^−1^) and the C–H bending (731 cm^−1^) were clearly observed. Besides the organic fragments, the Cu–Cu vibration band was observed at 480 cm^−1^ from the Raman spectrum in Fig. 2D, indicating homoatomic metal bonding. All these characteristic peaks were matched well with those of HKUST-1 described in earlier work,^38^ which also confirmed the formation of HKUST-1 in this work.

In addition, the structure of the nanosheets was found stable in the growth, assembly and precipitation process at room temperature. And the morphology remained unchanged even after prolonged centrifugal separation and ultrasonic oscillation.

**Fig. 3:**
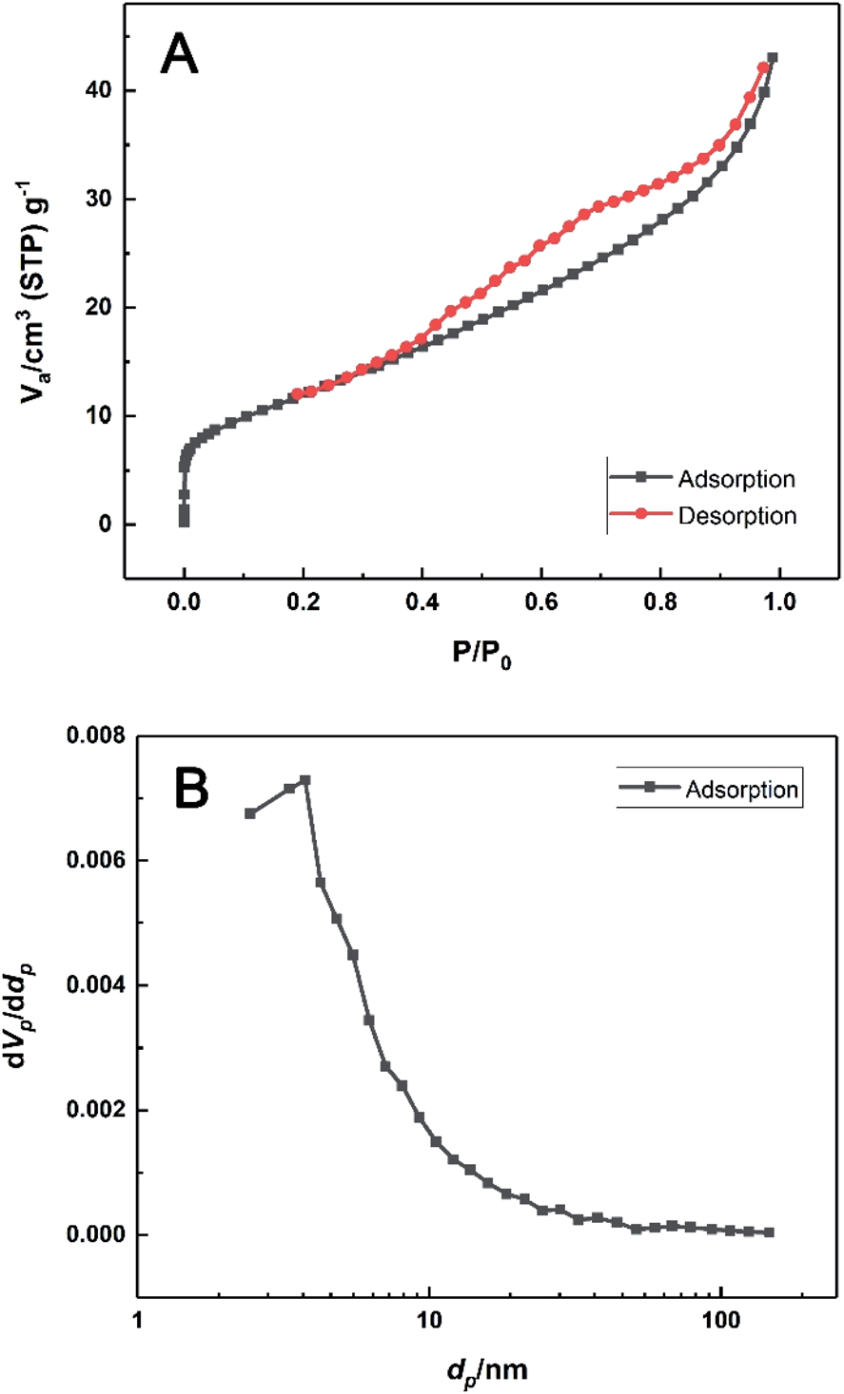
(A) N_2_ adsorption–desorption isotherms and (B) the BJH desorption pore size distribution of the prepared HKUST-1.

Fig. 3 displays the nitrogen adsorption–desorption isotherms of the material at 77 K. Clearly, the monolayer coverage was completed within the vicinity of the inflexion point in the low relative pressure region. Afterwards, the isotherm indicated infinite multilayer adsorption and no saturation limit was observed. This behaviour was typically an IUPAC Type II isotherm and the multilayer adsorption was attributed to the nanosheets structure of HKUST-1. The desorption isotherm presented a hysteresis loop of H4 type in the high-pressure region.^43^ Moreover, the BET specific surface area was determined as 45 m^2^ g^−1^, with the mean pore diameter of 5.92 nm, which was might resulted from sheet stacking or capillary condensation in the mesopores.

### 3.3. Electrochemical behaviour of HKUST-1

**Fig. 4:**
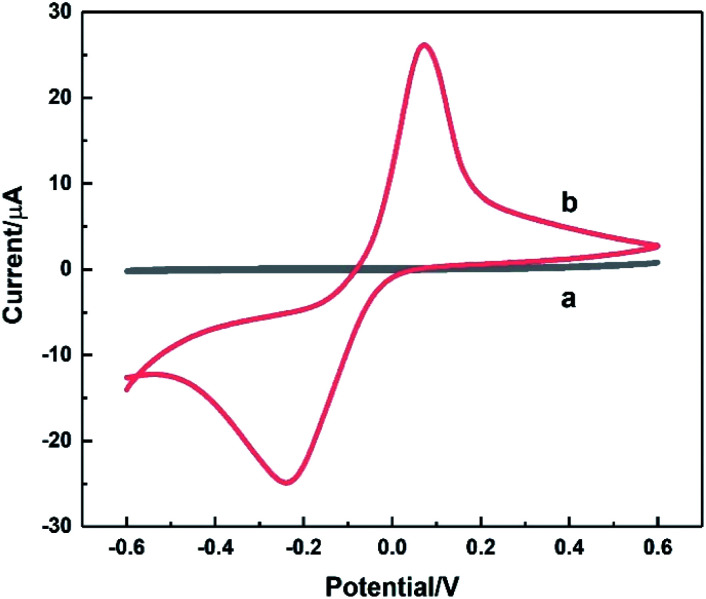
CV curves recorded on bare ITO (a) and HKUST-1/ITO (b) in 0.1 M pH 3.63 PBS at the scan rate of 100 mV s^−1^.

The electrochemical behaviour of HKUST-1/ITO was studied using CV before it was implemented on the detection of AA. In Fig. 4, the CV responses on ITO (curve a) and HKUST-1/ITO (curve b) in 0.1 M pH 3.63 PBS were recorded at a scan rate of 100 mV s^−1^. There were no obvious peaks observed on bare ITO due to non-electroactive material existed electrode surface. However, a pair of obvious oxidation–reduction peaks was observed on HKUST-1/ITO, corresponding to the conversion between Cu(ii) and Cu(i).^44^ Both the metallic Cu ion centre in HKUST-1 and the space between nanosheets enhanced the electron transfer greatly, improving the current dramatically.

### 3.4. Determination of effective surface area

**Fig. 5:**
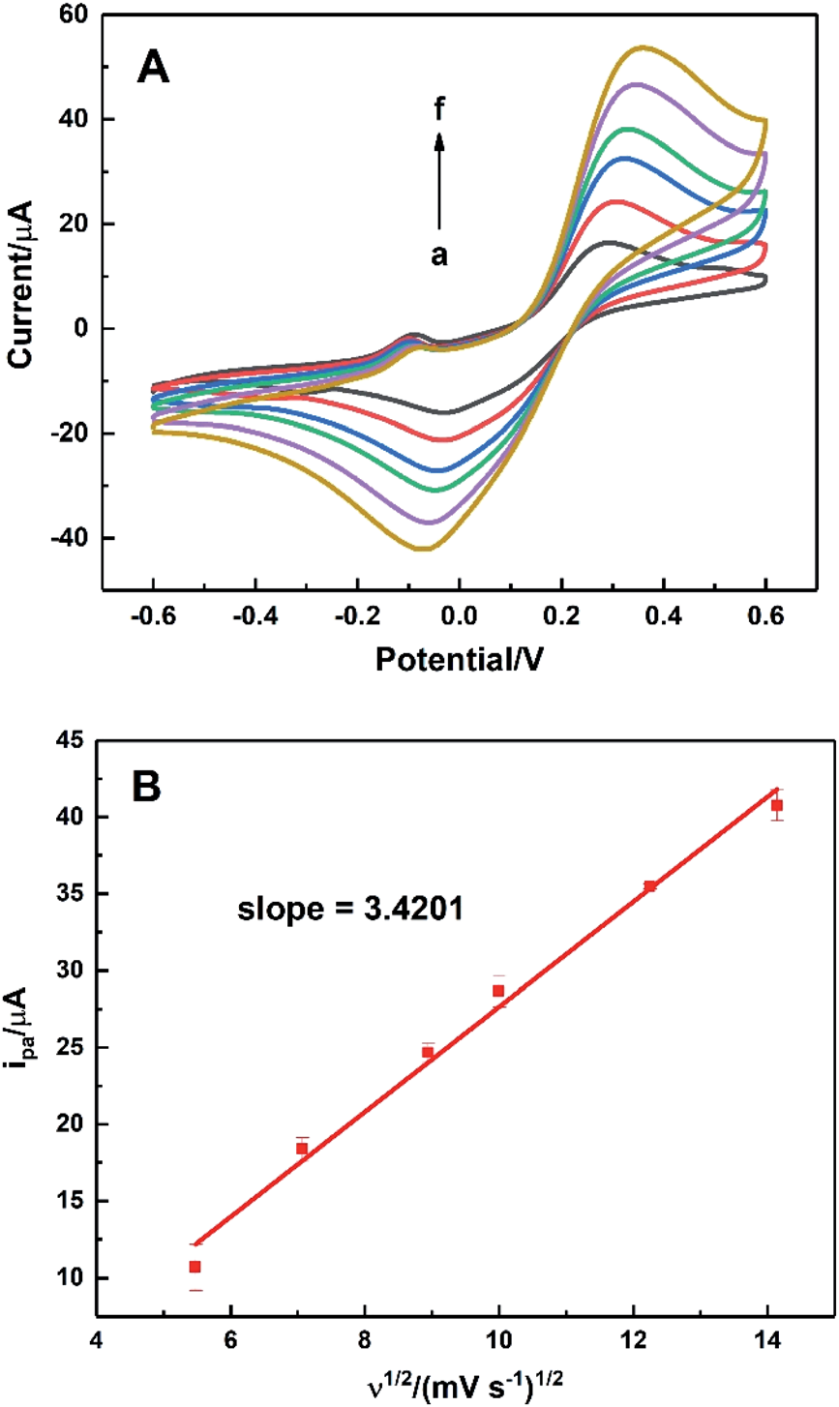
(A) CVs of HKUST-1/ITO in 2.5 mM K_3_Fe(CN)_6_/K_4_Fe(CN)_6_ solution containing 0.1 M KCl without AA at different scan rate of 30, 50, 80, 100, 150 and 200 mV s^−1^ (from a to f), (B) the linear plot of *i*_pa_*vs. ν*^1/2^.

To evaluate the effective surface area (ESA) of the HKUST-1/ITO electrode, CV analysis was applied in the redox system of Fe(CN)_6_^3−^/Fe(CN)_6_^4−^. Fig. 5A illustrated the CVs of HKUST-1/ITO in 2.5 mM K_3_Fe(CN)_6_ solution containing 0.1 M KCl at various scan rates. Then, the diffusion-controlled kinetics was also deduced from the linear relationship between oxidation peak currents *i*_pa_ and square root of scan rates *ν*^1/2^, as shown in Fig. 5B, with the regression equation of *i*_pa_/μA = 3.4201*ν*^1/2^/(mV s^−1^)^1/2^ − 6.5569 (*R*^2^ = 0.9913). Herein, the ESA value of the modified electrode could be calculated by Randles–Sevcik equation:^45,46^
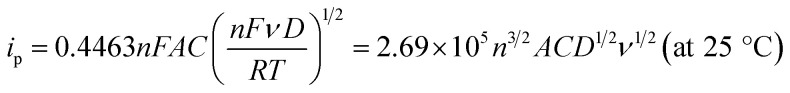
where *i*_p_ (A) is the peak current in an oxidation or reduction process, *n* = 1 is the total number of electrons exchanged in Fe(CN)_6_^3−^/Fe(CN)_6_^4−^ redox reaction, *A* (cm^2^) is the effective surface area, *C* (mol cm^−3^) is the concentration of the molecule Fe(CN)_6_^3−^ in the solution (2.5 mM), *D* (cm^2^ s^−1^) is the diffusion coefficient of Fe(CN)_6_^3−^ (6.5 × 10^−6^ cm^2^ s^−1^) and *ν* (V s^−1^) is the scan rate. In this case, the slope was 3.4201, hence the ESA of HKUST-1/ITO was determined as 0.06308 cm^2^, which was about 8.03 times of that of bare ITO. This indicated that the HKUST-1 modification enhanced the catalytic ability by providing more electrochemical active sites.

### 3.5. Electrocatalytic oxidation of AA on HKUST-1/ITO

**Fig. 6:**
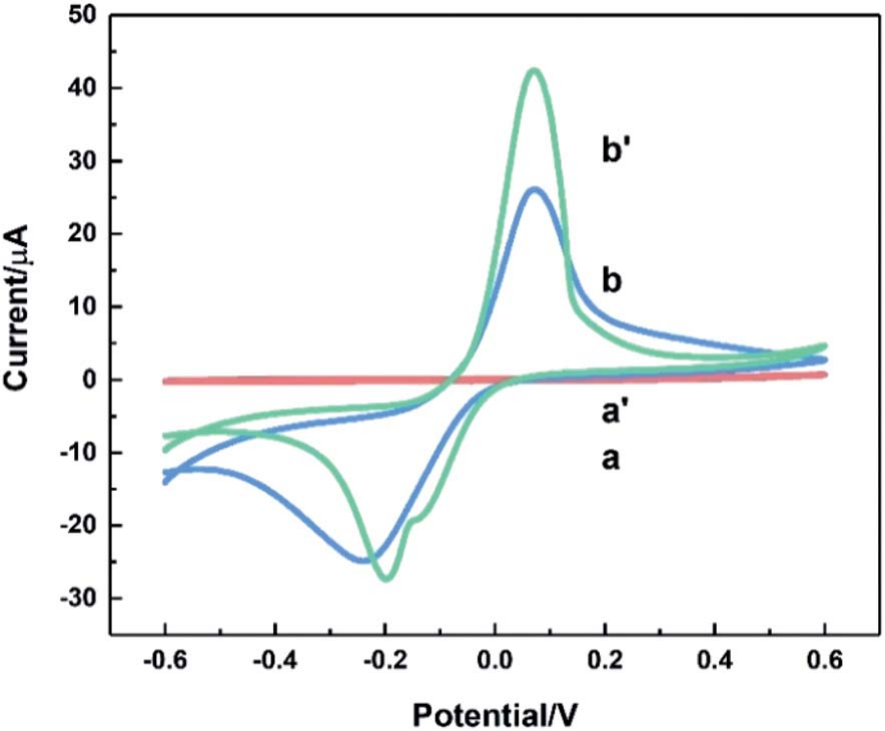
CVs of bare ITO (a and a′) and HKUST-1/ITO (b and b′) in 0.1 M pH 3.63 PBS solution without (a and b) and with 200 mM AA (a′ and b′) at the scan rate of 100 mV s^−1^.

Fig. 6 depicted CV curves on bare ITO (a and a′) and HKUST-1/ITO (b and b′) in 0.1 M pH 3.63 PBS in the absence (a and b) and presence (a′ and b′) of 200 mM AA at the scan rate of 100 mV s^−1^. Comparison of curve a and a′ revealed no evidence for the catalytic effect of bare ITO on the AA oxidation. However, from curve b and b′, the oxidation peak current increased about twofold towards AA, illustrating AA could be catalytically oxidised by HKUST-1. Furthermore, a possible mechanism focusing on the interaction between AA and HKUST-1 was investigated. AA was oxidised by Cu(ii), and meanwhile Cu(ii) was reduced to Cu(i). That is to say, HKUST-1 catalysed the oxidation of AA, and the oxidation peak current of HKUST-1 was enhanced. The overall reaction equations were as follows:^47,48^2Cu(ii) + C_6_H_8_O_6_ → 2Cu(i) + 2H^+^ + C_6_H_6_O_6_2Cu(i) → 2Cu(ii) + 2e^−^

### 3.6. Effect of solution pH

**Fig. 7:**
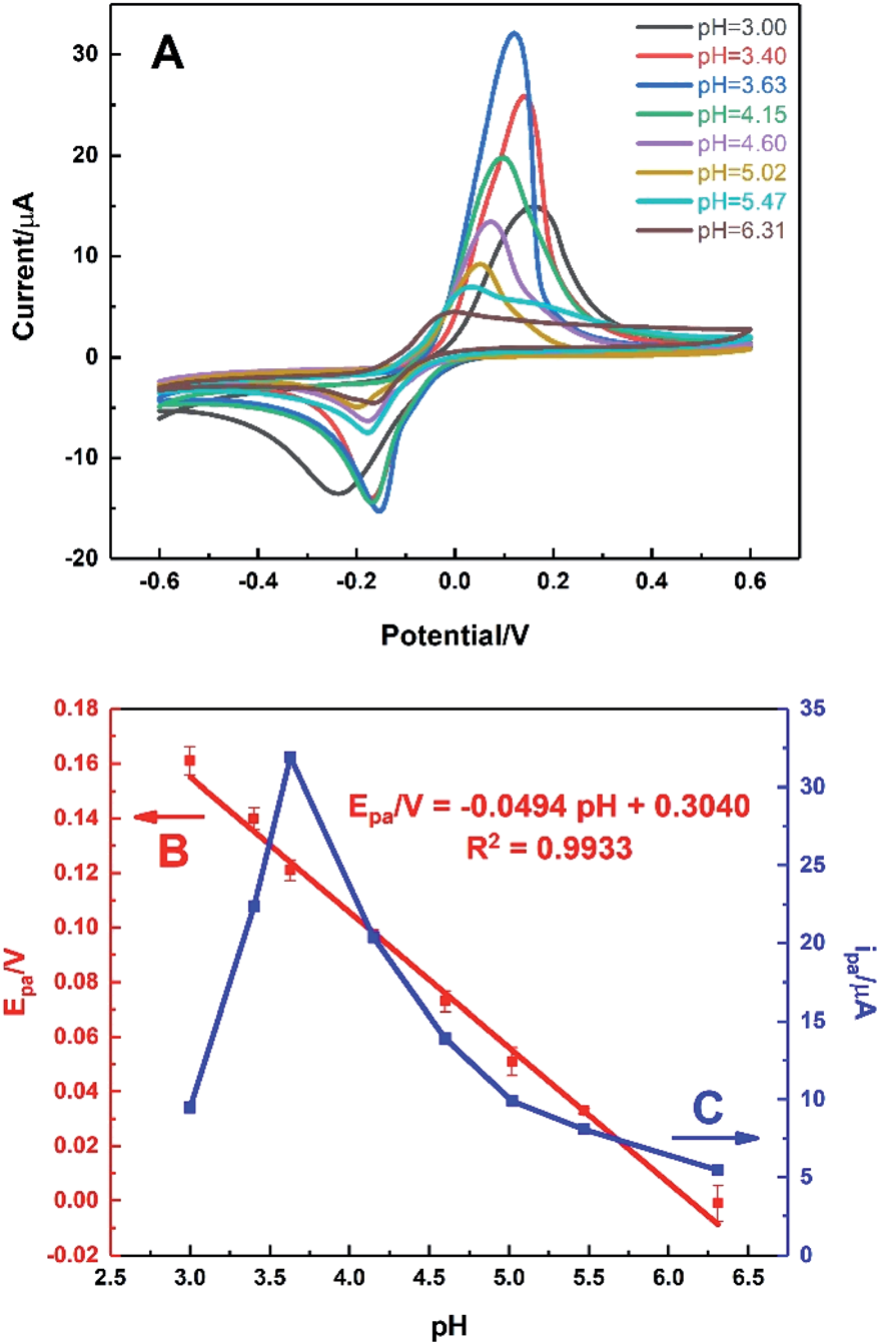
(A) CVs of HKUST-1/ITO in 0.1 M PBS solution under different pH values ranging from 3.00 to 6.31 for 1 mM AA at the scan rate of 100 mV s^−1^. Plots of (B) oxidation potential and (C) oxidation current *vs.* pH from CVs.

Fig. 7 shows the behaviour of HKUST-1/ITO in 0.1 M PBS solution at different pH values ranging from 3.00 to 6.31 in the presence of 1 mM AA *via* CV technique. It could be seen from Fig. 7A, the oxidation peak moved negatively as pH increased, illustrating it was a proton-participated electrochemical reaction. It was found that the oxidation potential of AA was inversely proportional to pH value (Fig. 7B), with a fitting equation of *E*_pa_/V = −0.0494pH + 0.3040 (*R*^2^ = 0.9933). It meant that AA could be oxidised at not only acidic but also neutral circumstances, indicating the further physiological application of the sensor. Moreover, the slope of −0.0494 V per pH unit was close to the theoretical value of −0.0592 V per pH unit in Nernst equation, illustrating the electron transfer process was accompanied by equal number of protons transferred. In Fig. 7C, it could be seen that the oxidation current of AA increased sharply from pH 3.00 to 3.63, achieving the highest value. Then, the current decreased rapidly as pH rose, which might be owing to the proton-participated reaction. Thus, the optimal pH value was chosen as 3.63 in this work to ensure the high sensitivity.

### 3.7. Effect of modification amount of HKUST-1

**Fig. 8:**
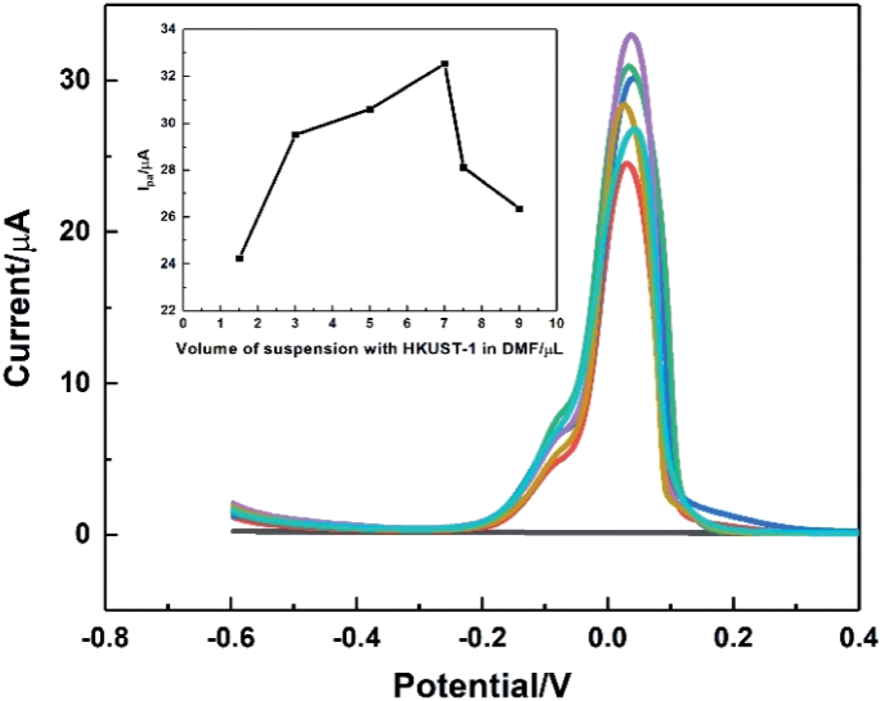
DPVs recorded on HKUST-1/ITO with different modification volume of 2 mg mL^−1^ HKUST-1 suspension from 1.5 to 9.0 μL, in 0.1 M pH 3.63 PBS solution upon 1 mM AA at the scan rate of 100 mV s^−1^. Inset was the plot of *I*_pa_*vs.* modification volume.

The influence of the modification amount of HKUST-1 was studied using DPV, and the result was shown in Fig. 8. The modification amount did have great impacts on the oxidation peak current. It was found that the oxidation current of AA increased sharply when the modification amount of 2 mg mL^−1^ HKUST-1 suspension increased from 1.5 to 7.0 μL. The possible reason might be that the large amount of catalyst brought enhanced catalytic ability. However, the current decreased as the modification amount more than 7.0 μL, attributing to the fact that the thicker modification layer was easy to crack and fall off from the electrode surface. Therefore, 7.0 μL suspension of HKUST-1 was selected as the optimal modification amount in this work.

### 3.8. Effect of scan rates on dynamics of AA oxidation

**Fig. 9:**
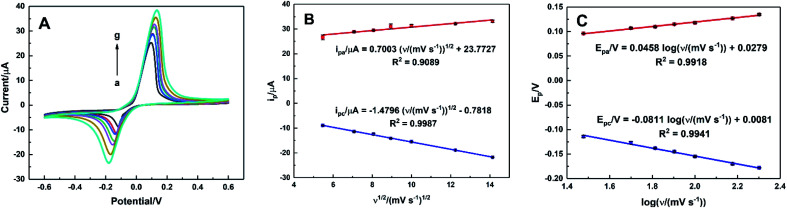
(A) CVs of HKUST-1/ITO in 0.1 M pH 3.63 PBS solution containing 1 mM AA at different scan rate of 30, 50, 65, 80, 100, 150 and 200 mV s^−1^ (from a to g), (B) the linear plots of *i*_pa_ and *i*_pc_*vs. ν*^1/2^, and (C) the linear plots of *E*_pa_ and *E*_pc_*vs.* log *ν*.

The effect of the scan rate on the electrocatalytic oxidation of AA was investigated by CVs of HKUST-1/ITO in 0.1 M pH 3.63 PBS solution containing 1 mM AA within the range of 30–200 mV s^−1^. As illustrated in Fig. 9A, the oxidation potential shifted positively and the reduction one shifted negatively, suggesting an irreversible electrochemical process. Meanwhile, both the oxidation and reduction peak currents increased as the scan rate increased. The relationships between the anodic and cathodic current and *ν*^1/2^, expressed in Fig. 9B, were *i*_pa_/μA = 0.7003*ν*^1/2^/(mV s^−1^)^1/2^ + 23.7727 (*R*^2^ = 0.9089) and *i*_pc_/μA = −1.4796*ν*^1/2^/(mV s^−1^)^1/2^ − 0.7818 (*R*^2^ = 0.9987), indicating a diffusion-controlled electrode process.

Fig. 9C showed the linear equations of the potential *versus* the logarithm of scan rate as *E*_pa_/V = 0.0458 log *ν*/(mV s^−1^) + 0.0279 (*R*^2^ = 0.9918) and *E*_pc_/V = −0.0811 log *ν*/(mV s^−1^) + 0.0081 (*R*^2^ = 0.9941), respectively. A plot of logarithmic current *vs.* potential, known as a Tafel plot, is one of the useful approaches to kinetic parameters. Generally, it takes the form of
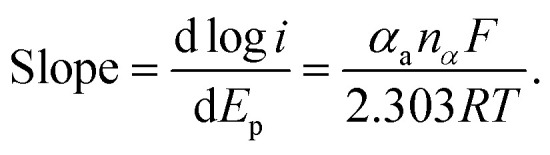


**Scheme 1:**
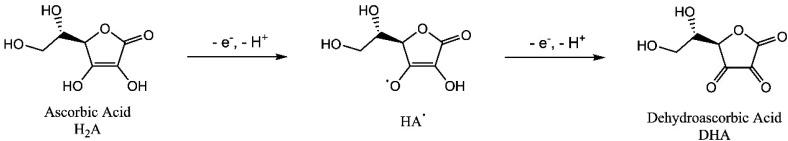
A feasible mechanism of ascorbic acid oxidation.

Herein, for the irreversible and diffusion-controlled system, the relationship of peak potential shifts towards scan rate could be deduced as the following equations:^46,49^
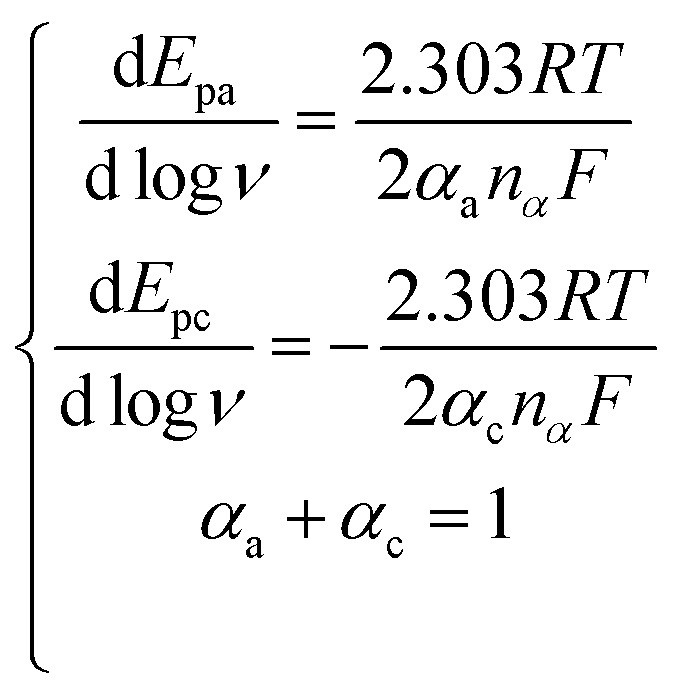
where *α*_a_ (*α*_c_) is the anodic (cathodic) charge transfer coefficient and *n*_*α*_ is the number of electrons transferred in the rate-limiting step. Then the dynamics-relating parameters could be calculated from the slopes of the plots as *α*_a_ = 0.64, *α*_c_ = 0.36 and *n*_*α*_ = 1.01, confirming the irreversible process and proving the rate of the oxidation was determined by the single electron transfer. Considering the process underwent an equal-electron-equal-proton pathway, a feasible mechanism of AA oxidation was proposed in Scheme 1.^50,51^

Moreover, with the dynamics-relating parameters, the diffusion coefficient of AA could be deduced from the modified Randles–Sevcik equation for irreversible processes:^46,52^
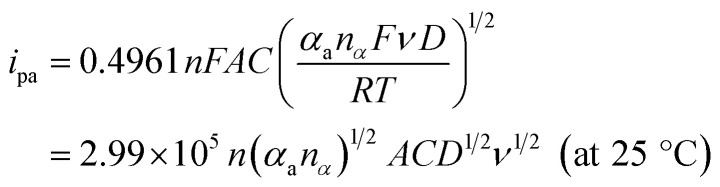
where *n* = 2 is the total number of electrons exchanged in AA oxidation, *A* = 0.06308 cm^2^ is the effective surface area calculated before, and all the various parameters are with the same meanings and in the same units as mentioned above. According to the slope of Fig. 9B, the diffusion coefficient was estimated to be 5.33 × 10^−7^ cm^2^ s^−1^, which corresponded to the value reported previously.^53,54^

### 3.9. Electrochemical detection of AA

**Fig. 10:**
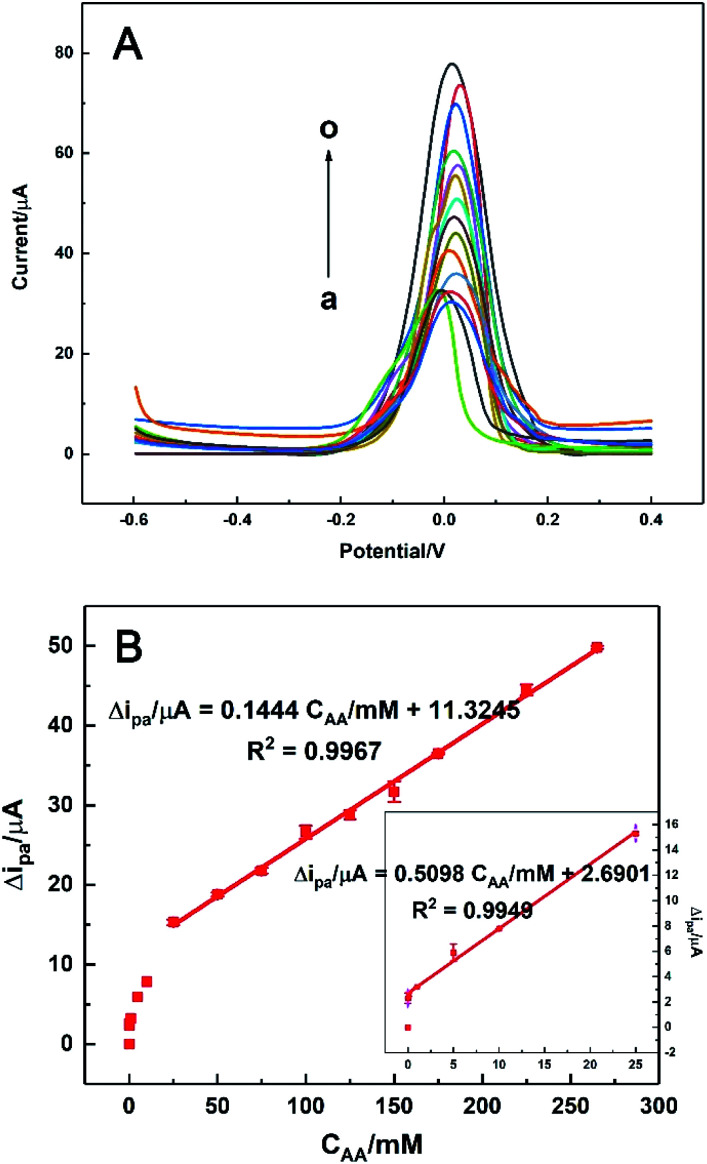
(A) DPVs of HKUST-1/ITO in 0.1 M pH 3.63 PBS solution with different concentrations of AA at scan rate of 100 mV s^−1^: 0, 0.01, 0.1, 1, 5, 10, 25, 50, 75, 100, 125, 150, 175, 225 and 265 mM (from a to o). (B) The calibration curve of Δ*i*_pa_*vs. C*_AA_ at high concentrations and lower concentrations (inset), respectively.

Under the optimal conditions, the electrocatalytic responses of AA with different concentrations on HKUST-1/ITO were determined using DPV technique. As shown in Fig. 10A, the oxidation current increased when AA was added successively from 0.01 to 265 mM, while the peak potential kept around +0.02 V. The linear range of Δ*i*_pa_*vs. C*_AA_ was composed of two parts (Fig. 10B). Here, Δ*i* was the difference between *i*_p_ and *i*_0_, corresponding to the current responses with and without AA in solution, respectively. One linear part was at a lower concentration range of 0.01–25 mM, where the current grew rapidly and the regression equation was Δ*i*/μA = 0.5098*C*_AA_/mM + 2.6901 (*R*^2^ = 0.9949, *n* = 6). The other linear part was at a higher concentration range of 25–265 mM, but the current increased in a smaller slope than before, with the linear relationship was Δ*i*/μA = 0.1444*C*_AA_/mM + 11.3245 (*R*^2^ = 0.9967, *n* = 6). The sensitivity in the higher concentration range was about 28% of that in the lower concentration range, implying the catalytic rate became decreased in this situation. Furthermore, the detection limit was estimated as 3 μM according to S/N = 3. The sensing performance of the proposed AA sensor was also compared with those in previously reported works, as listed in Table 1. It could be seen that our sensor revealed a wider linear range and a lower detection limit, which might since the high ESA of HKUST-1 modification provided more active sites available for electrocatalysis and the nanosheet framework structure of HKUST-1 accelerated the electron transfer between AA and electrode surface.^19,47,55–62^

**Table 1:** Comparison of the proposed AA sensor performance with those in previous works

AA sensor	Linear range/mM	Detection limit/μM	Ref.
Cu_4_(OH)_6_SO_4_/ITO	0.017–6	6.4	47
Pre-anodised CPE	0.01–1.5	0.31	19
AuNPs@MoS_2_/GCE	0.05–100	50	55
GEF/CFE	0.07–2.31	73.52	56
ZnO–Cu_*x*_O–PPy/GCE	0.2–1	25.0	57
rGO-CNT/ITO	0.01–0.2	5.31	58
Pd@Au/rGO/GCE	0.05–2.86	24.88	59
AuNPs/PDDA/GNS/GCE	0.6–4.2	80	60
HNP-PtTi/GCE	0.2–1	24.2	61
PrGO/PB/GCE	0.283–2.33	34.7	62
HKUST-1/ITO	0.01–25 and 25–265	3	This work

### 3.10. Repeatability, stability and selectivity of the sensor

The repeatability was assessed using one working electrode for 15 continuous measurements towards 1.0 mM AA in 0.1 M pH 3.63 PBS solution at the scan rate of 100 mV s^−1^. The relative standard deviation (RSD) of the tested data was 2.18%, showing favourable repeatability. Additionally, the short-time and long-time stability of the sensor were tested within a period of 7 and 21 days, respectively after its preparation. During this time, the modified electrodes were stored in 0.1 mM pH 3.63 PBS solution. Only 2.17% and 6.91% of the current responses were found to decrease, respectively, thus indicating good stability of the sensor. Both the good repeatability and stability were attributed to the stable structure of HKUST-1 modification. In addition, it is also found that the prepared HKUST-1 is still stable towards acidic circumstance, which is consistent with the previous research.^63^

**Fig. 11:**
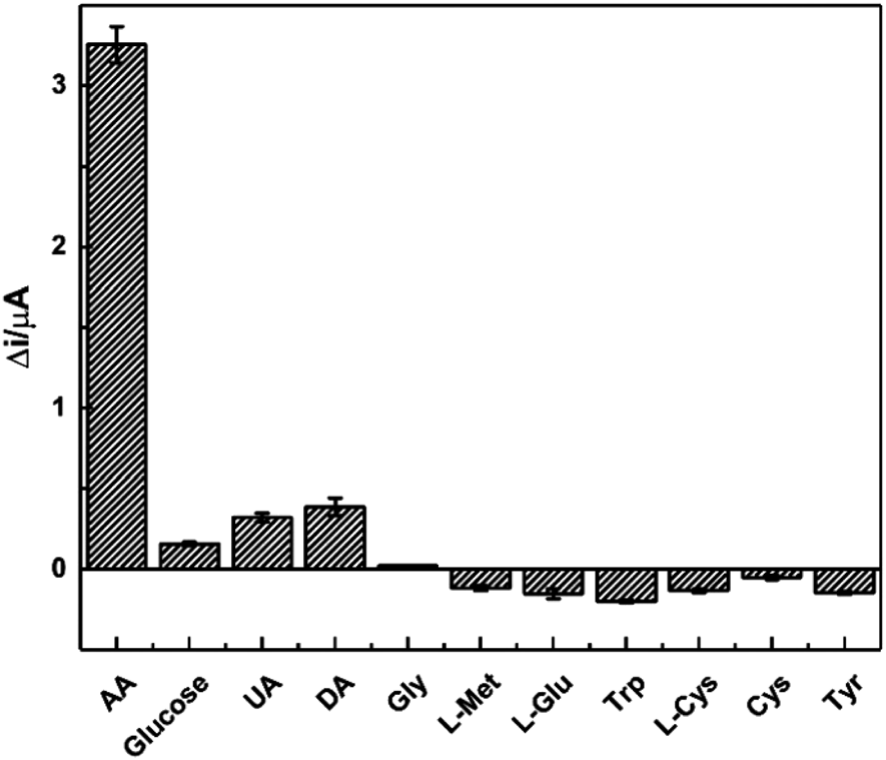
Plot of Δ*i* responses towards 1.0 mM of AA and ten interfering substances of glucose, UA, DA, Gly, l-Met, l-Glu, Trp, l-Cys, Cys and Tyr.

The selectivity plays an essential role in the detection of real samples and a common way was utilised to investigate the selectivity of the sensor. Ten different interfering substances, including glucose, DA, UA, and amino acids, *etc.*, were examined at the concentration of 1.0 mM in PBS solution, and the results were shown in Fig. 11. The current responses from glucose, UA and DA were less than 10% of that from AA, and most of the amino acids just reduced the current slightly. This certifies the high selectivity of the AA sensor, which forms the basis for further practical application.

### 3.11. Analytical application on real samples

In order to evaluate the practical performance of the novel AA sensor with HKUST-1/ITO, the proposed method was employed to detect the AA content in various healthcare tablets purchased from market. The tablets were dissolved in pH 6.5 PBS first, and the pH value of the solution was adjusted to 3.63 by adding 0.2 M H_3_PO_4_ after filtrating the insolubilities. The results were listed in Table 2. All of them were examined in 0.1 M pH 3.63 PBS solution at room temperature and repeated for three times, the obtained relative error (RE) values compared with the nominal values outside the commodity packages were all lower than ±3%, indicating accurate detection in real sensing system and verifying the excellent selectivity of the sensor.

**Table 2:** Determination of AA in real tablets

Sample	Sample concentration/mM	Measured concentration/mM	RE
Vitamin C pill	11.24	11.37	+1.15%
Vitamin C buccal tablet	13.63	13.56	−0.49%
Multi-vitamin effervescent tablet	42.58	43.68	+2.57%

## 4. Conclusions

In summary, a typical type of Cu-based nanosheet-structure MOFs (HKUST-1) nanomaterials was prepared in this work, and based on which a novel electrochemical sensor for AA detection was fabricated. HKUST-1 exhibited good electrocatalysis to AA, since its porous structure promoted the electron transfer significantly. Besides, a two-step mechanism was proposed to explain the oxidation pathway of AA, where the equal number of electrons and protons were involved. Under the optimal experimental conditions, the AA sensor exhibited a wide linear range of 0.01–25 and 25–265 mM, a low limit of detection of 3 μM at S/N = 3, good reproducibility, favourable stability and high sensitivity. Furthermore, it was successfully applied in the real sample testing, indicating potential practical value.

## Conflicts of interest

There are no conflicts to declare.

## Supplementary Material
